# Serum and CSF cytokine profile in patients with facial palsy (Bell’s palsy): a pilot study

**DOI:** 10.1007/s00415-025-13561-8

**Published:** 2025-12-09

**Authors:** Alexander Ustinov, Josef Georg Heckmann, Vijay Singh, Borros Arneth, Stefan Schwab

**Affiliations:** 1 Department of Neurology, Medical Campus Lower Bavaria, Hospital Landshut, Robert-Koch Str. 1, 84024 Landshut, Germany; 2 Department of Neurological Rehabilitation, Asklepios Klinik Schaufling, 94571 Schaufling, Germany; 3 Faculty of Medicine, University Erlangen-Nuremberg, 91054 Erlangen, Germany; 4 Department of Pediatric Hematology and Oncology, University Hospital Giessen and Marburg, Justus Liebig University Giessen, 35382 Giessen, Germany; 5 Institute of Laboratory Medicine and Pathobiochemistry, University Hospital Giessen and Marburg, Justus Liebig University Giessen, 35382 Giessen, Germany; 6 Department of Neurology, University Hospital Erlangen, 91054 Erlangen, Germany

**Keywords:** Facial palsy, Bell’s palsy, Cytokines, Cerebrospinal fluid

## Abstract

**Background and purpose:**

Facial nerve palsy is the most common cranial nerve disorder, and over 60% are idiopathic (Bell’s palsy, BP). An inflammatory process is discussed as a causative factor. The aim of this study was to search for changes in various cytokine concentrations in serum and cerebrospinal fluid (CSF) in patients with facial nerve palsy.

**Methods:**

In this prospective study, 47 patients with peripheral facial nerve palsy were included. Of these, 40 patients were diagnosed as BP and seven patients as non-idiopathic peripheral facial palsy (nipFP). Seventeen cytokines, including IL-1A, IL-5, IL-9, granulocyte colony-stimulating factor (G-CSF), CXCL-13, granulocyte–macrophage-colony-stimulating factor (GM-CSF), tumor necrosis factor-SF13 (TNFSF13), IL-8, IL-1ß, CXCL-10, fractalkine (Fract), monocyte chemotactic protein (MCP-1), IFN-y, IL-4, IL-17A, tumor necrosis factor (TNF), and Granzyme, were measured in the serum and CSF. For comparison, sera from 10 healthy individuals were used.

**Results:**

In serum, the levels of cytokines G-CSF, CXCL13, TNFSF13, and Granzyme were statistically significantly higher in patients with BP and nipFP compared to healthy individuals (*p* < 0.05). Cytokine IL-1ß was significantly higher in nipFP patients compared to healthy individuals and patients with BP (*p* < 0.05). Cytokine IL-8 was significantly lower in both patient groups than in healthy individuals (*p* < 0.05). In CSF, G-CSF, CXCL13, TNFSF13, IL-8, IL-1ß, and IL-17A were statistically significantly higher in patients with nipFP compared to patients with BP (*p* < 0.05). In addition, patients with BP also showed a clustering of cytokine elevation. For differentiating nipFP from BP, serum G-CSF and IL1ß indicated a certain discrimination (AUC 0.643; 0.614). Patients with severe facial palsy revealed higher CSF TNFSF13 (*p* = 0.02), and clinical outcome after 3 months was less favorable at higher CSF Fractalkine (*p* = 0.025). Elevated CSF cell count was associated with elevated CXCL13, IL-8, IL-1ß, IP-10, IFNa and granzyme in the CSF (*r* = 0.32–0.57; *p* < 0.05).

**Conclusion:**

Compared to healthy individuals, our study revealed an altered cytokine profile in patients with BP that resembles patients with nipFP. In CSF, a subset of cytokines was identified in patients with BP, but higher levels were found in patients with nipFP, suggesting a graduated inflammatory process.

**Study registration:**

The study "Serum and CSF cytokine profile in patients with facial palsy (Bell´s palsy)" has been officially registered at the German Clinical Trials Register (DRKS00037815).

## Introduction

Facial nerve palsy is the most common cranial nerve disorder. The idiopathic form (Bell’s palsy = BP) accounts for 60–75% of acquired peripheral facial nerve palsies. It occurs in 7–40 patients per year and 100,000 inhabitants, with men and women being affected about equally [1]. Pathophysiologically, the reactivation of a herpes simplex virus infection (HSV type I) and the cell-mediated autoimmune inflammation are currently being discussed as the most important causative factors. This viral hypothesis is supported by the detection of HSV nucleic acid in the geniculate ganglia or in the endo-neuronal fluid obtained during decompression surgery and the detection of HSV genome in the saliva of affected patients. Similar to herpes labialis, a provocation by stress without concomitant infection would then be regarded as the triggering factor. However, objections against the viral hypothesis are the lack of mucocutaneous changes and the almost non-occurring recurrences as is the case with herpes labialis, for example [2, 3].

In contrast, the autoimmune hypothesis is referred to as a mononeuritic variant of Guillain–Barré syndrome. This concept is supported by findings of a reduction in T suppression cells and an increase in T lymphocytes in affected patients as well as the increased levels of interleukin 1, interleukin 6, and tumor necrosis factor (TNF) alpha in the serum. It is interesting to note the epidemiological observation that the frequency of idiopathic facial nerve palsy was significantly increased after nasal vaccination against influenza in Switzerland [4]. Slightly higher incidences were also observed following infection with the coronavirus and after vaccination against SARS-CoV-2 [5, 6]. The hypothesis would be that such a mononeuritic immune response is triggered after an infection (or vaccination), for example, which is directed against peripheral nerve myelin antigens.

Cytokines are peptides of around 100–200 amino acids and act as mediators to trigger cell- and cytokine-specific signals. They can have both pro- and anti-inflammatory effects and are involved in controlling the immune response. From a regulatory point of view, it is important that cytokine receptors are not only found on immunocompetent cells and hormone-producing cells but also on nerve cells [7]. Moreover, cytokines can exert their effect even in the absence of an infection [8]. In pathological conditions, the cellular expression profiles and concentrations of individual cytokines are significantly altered. Studies on cytokine levels in serum and cerebrospinal fluid (CSF) in patients with facial nerve palsy are sparse to date [9–11]. The aim of this prospective study was to search for changes in various cytokine concentrations in serum and CSF in patients with facial nerve palsy.

## Methods

### Study design and ethics

This prospective study was conducted at the Neurological Department of Landshut Hospital (Medical Campus Lower Bavaria, Germany) in accordance with the Declaration of Helsinki and was reviewed and approved by the Ethics Committee of Landshut Hospital (decision of the Ethics Committee of 21 March 2022). This study was subsequently registered at the German Clinical Trials Registry (Deutsches Register für Klinische Studien, DRKS00037815). All patients received oral information and an information letter about the study, the data analysis, and the option to decline participation in the study. Written informed consent was necessary to participate in the study. All data were anonymized.

All adult patients who presented to the emergency department of Landshut Hospital with non-traumatic peripheral facial nerve palsy between April 2022 and April 2024 were screened for the study. Patients were included if they had an initial diagnosis of presumed idiopathic facial nerve palsy in the emergency department. The patients were subsequently diagnosed and treated according to the guidelines of the German Society of Neurology for the diagnosis and treatment of idiopathic facial nerve palsy [12]. The exclusion criteria for the current study included the following: younger than 18 years, sequelae of facial nerve palsy from a previous disease, bilateral facial palsy, initial diagnoses other than suspected peripheral idiopathic facial nerve palsy, refusal of lumbar puncture, and refusal to participate in the study.

As soon as a patient was selected and gave his consent, the additional serum and the CSF samples taken were sent to the Institute of Laboratory Medicine at Giessen-Marburg University Hospital (Germany) for further analysis. The severity of the facial nerve palsy in the patient was categorized according to the House–Brackmann (HB) scale ranging from grade I (no palsy) to grade VI (complete palsy). Grade III refers to moderate dysfunction with still barely possible lid closure. In grade IV lid closure is insufficiently [1]. All patients were clinically re-examined after 3 months. The following characteristics were collected: demographic characteristics, clinical severity of facial palsy, routine laboratory data, and judgment on improvement at follow-up, which was carried out by either outpatient visit or by telephone. Patients were classified as (a) having a confirmed idiopathic facial nerve palsy (BP) if the CSF was normal (cell count < 5cells/µl and protein < 450 mg/L) and no other cause was considered, or (b) having a non-idiopathic peripheral facial nerve palsy (nipFP) if the initial diagnosis had to be corrected based on CSF findings (increased cell count and increased protein, abnormal antibody specificity index, pathogen detection using PCR) and additional findings (abnormal clinical and imaging findings) [1, 13]. The sera of healthy individuals were used for comparison purposes.

### Determination of cytokines

In selecting analytes, we aimed to probe complementary immune axes relevant to facial nerve inflammation and to differentiate infection-driven from autoimmune neuritic signatures. To achieve this, we designed two customized cytokine panels that captured distinct yet complementary aspects of the immune response associated with facial nerve palsy. Panel 1 focused on cytokines involved in innate myeloid activation, granulopoiesis, and B cell chemoattraction (G-CSF, GM-CSF, CXCL13, TNFSF13, IL-1A, IL-5, IL-9). Panel 2 targeted cytokines linked to proinflammatory signaling, T helper cell polarization (Th1/Th2/Th17), chemokine-mediated leukocyte recruitment, and cytotoxic effector activity (IL-1β, IL-8, IP-10, Fractalkine, MCP-1, IFN-γ, IL-4, IL-17A, TNF, Granzyme A). Together, these panels enabled interrogation of both innate and adaptive immune pathways, with the overarching goal of identifying pathogenic clues regarding cell populations and immune mechanisms active in BP versus nipFP.

Serum and CSF samples from patients were collected within 72 h after admission and sent to the laboratory frozen. The samples were thawed overnight at 4 °C prior to analysis and quantified in two customized panels.

In Panel 1, concentrations of IL‑1A, IL‑5, IL‑9, G‑CSF, CXCL13, GM‑CSF, and TNFSF13 were measured in serum and CSF using the BioLegend LEGENDplex™ Multi‑Analyte Flow Assay Kit (Custom Human Assay) following the manufacturer’s protocol. Lyophilized recombinant cytokine standards were reconstituted and serially diluted into seven concentrations to generate standard curves. Standards and samples were run in duplicate. Each 25 µL sample was incubated with 25 µL of pre‑mixed antibody‑labeled beads for 2 h at room temperature in dark, followed by sequential incubations with 25 µL detection antibody for 1 h and 25 µL streptavidin–PE for 30 min in dark. After washing, samples were analyzed on a BD FACSLyric flow cytometer (BD, Heidelberg), and cytokine concentrations were calculated from standard curves using BioLegend LEGENDplex Data Analysis Software (Qognit Inc.).

In Panel 2, IL‑8, IL‑1β, IP‑10, Fractalkine, MCP‑1, IFN‑γ, IL‑4, IL‑17A, TNF, and Granzyme A were quantified in serum and CSF using the Human Enhanced Sensitivity Flex Sets (BD Biosciences, USA) according to the manufacturer’s instructions. Lyophilized recombinant cytokine standards were reconstituted and serially diluted into nine concentrations to generate standard curves. Like before, standards and samples were analyzed in duplicate. For each assay, 50 µL capture beads were mixed with 50 µL of sample or standard and incubated for 1 h at room temperature in the dark, followed by addition of 50 µL PE‑labeled detection antibodies and a further 2 h incubation. Following incubation, samples were washed with wash buffer to remove unbound reagents and analyzed on a BD FACSLyric instrument. Bead fluorescence data were processed using BD FCAP Array v3 software, and analyte concentrations were calculated from the standard curves as recommended by the manufacturer.

### Pilot study endpoints

The primary endpoint of the study was to compare cytokine levels in serum in patients with BP, nipFP and healthy individuals and in CSF in patients with BP and nipFP. Secondary endpoints were a correlation matrix of the analyzed cytokines, evaluation of the diagnostic area under the curve (AUC) of the cytokines using receiver operating characteristics (ROC) curves, correlation with severity and clinical outcome after 3 months and correlation of cytokines with CSF cell count.

### Statistical analysis

Statistical analysis was performed using NUMIQO (2025), a commercially available statistics program (numiqo: Online Statistics Calculator. numiqo e.U. Graz, Austria. URL https://numiqo.de). To test whether one or more categories of a contingency table had a statistically significant difference, the chi-square test, the Levene’s test, and the Fisher’s exact test were performed, depending on the statistical condition. For comparison of two or more groups’ parametric t tests, analysis of variance (ANOVA), nonparametric Mann–Whitney U test, Kruskal–Wallis test, and post hoc Bonferroni test or Fisher’s least significant test were used depending on the statistical conditions [14]. The non-parametric Spearman test was used for the correlation analysis. The AUC of the ROC was used to evaluate diagnostic accuracy of serum cytokines to differentiate between nipFP and BP. *P* value ≤ 0.05 indicated statistical significance.

## Results

### Population demographics

**Fig. 1:**
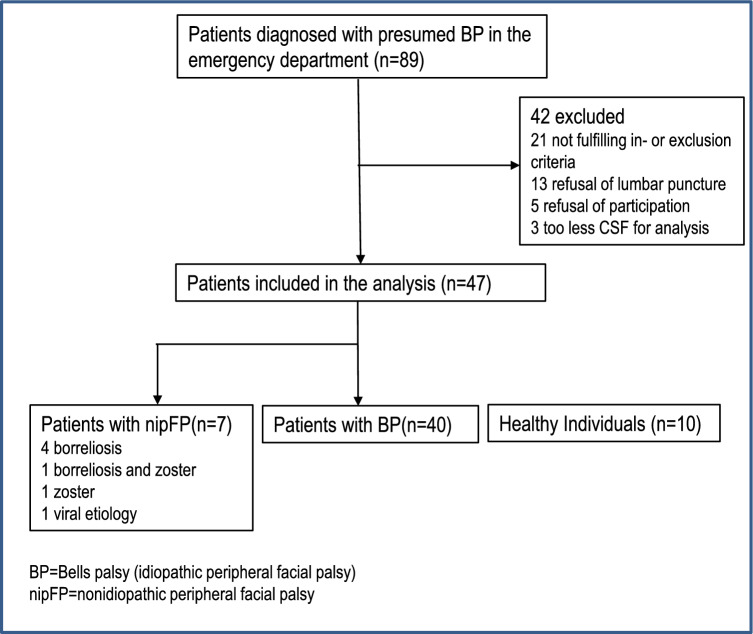
Study flow chart

Initially, 89 patients presented to the emergency department with peripheral facial nerve palsy between April, 2022 and April, 2024. Of these, 47 (53%) were diagnosed with presumed idiopathic facial nerve palsy (BP) during the initial consultation (emergency department visit). Among these patients, seven patients (15%) had non-idiopathic peripheral facial palsy (nipFP) because the initial diagnosis was corrected by the laboratory findings (four patients had neuroborreliosis, one patient had a combined infection with *Borrelia* and Varicella zoster, one patient had an isolated Varicella zoster infection, and one patient had viral facial paralysis caused by coronavirus). Figure 1 shows the enrollment of the study participants.

The study finally included 47 patients with a mean age at diagnosis of 57 (SD 27) years. 24 patients (51%) were women and 23 patients (49%) were men. Overall, 31 had right-sided facial nerve palsy and 16 left-sided facial palsy, 22 (48%) had mild peripheral facial nerve palsy (HB grades II–III, indicating eye closure possible), and 24 patients (52%) had severe peripheral facial nerve palsy (HB grades IV–VI, indicating no eye closure possible). The characteristics of the patients regarding the final diagnosis (BP versus nipFP) are shown in Table 1. With regard to gender, age, affected side, initial severity of facial palsy, presence of diabetes mellitus, symptoms at follow-up, CRP, and glucose and lactate of CSF, there was no statistically significant difference between the two patient groups. Patients with nipFP had significantly higher cell count and protein in CSF (*p* = 0.002; *p* = 0.007) than patients with BP.

**Table 1:** Clinical characteristics of patients with peripheral facial palsy

	Patient group		
	Confirmed BP	nipFP	
	(*n* = 40)	(*n* = 7)	*P* value
Gender			
Women	23 (58%)	1 (14%)	0.47
Men	17 (42%)	6 (86%)	
Age, mean (SD), y	57 (27)	68 (6)	0.38
Affected side			
Right	26 (65%)	5 (71%)	1
Left	14 (35%)	2 (29%)	
Initial HB grade^a^			0.42
II–III	20 (51%)	2 (29%)	
IV–VI	19 (49%)	5 (71%)	
Type 2 diabetes	8 (20%)	0 (0%)	0.33
Symptoms at 3 months			
Improvement	30 (75%)	5 (71%)	0.63
No change	2 (5%)	1 (14%)	
Undetermined	8 (20%)	1 (14%)	
CRP, mean (SD) (mg/dL)^b^	0.24 (0.28)	0.42 (0.41)	0.09
Cell count CSF, mean (SD) (/µl)^c^	2.6 (2.8)	62 (86)	0.002
Protein CSF, mean (SD) (mg/L)^d^	383 (226)	685 (541)	0.007
Glucose CSF, mean (SD) (mg/dL)^e^	71 (21)	57 (15)	0.105
Lactate CSF, mean (SD) (mmol/L)^f^	1.8 (0.4)	2.2 (1.2)	0.71

^a^Assed in 39 patients in the confirmed BP group

^b^Normal value < 0.5 mg/dL

^c^Normal value < 5/µl

^d^Normal value < 450 mg/dL

^e^Normal value 40–80 mg/dL

^f^Normal value 1.2–2.1 mmol/L

*SD* Standard deviation, *y* Year, *HB grade* House–Brackmann grade, *BP* Bell’s palsy, *nipFP* Non-idiopathic peripheral facial palsy

For analysis of cytokines, sera from healthy individuals (*n* = 10, 5 men, 5 women, mean age 28.1 years, SD 1) were used for comparison purposes.

### Characteristics of cytokines in the serum

**Fig. 2:**
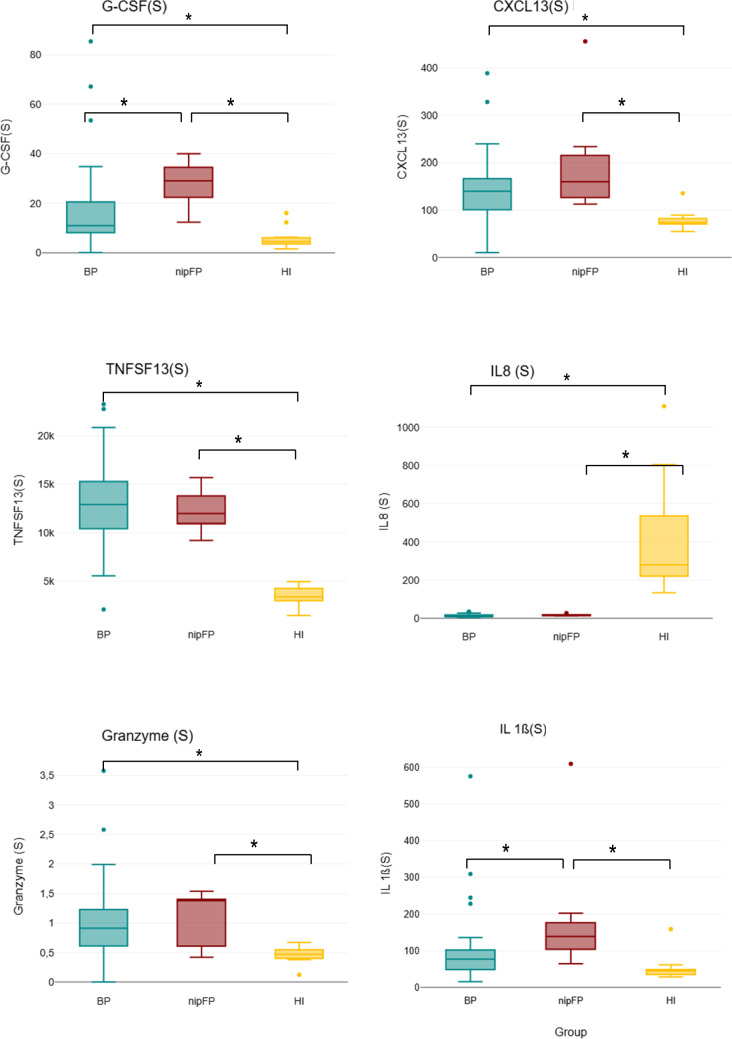
Serum (S) cytokine levels in patients with peripheral facial nerve palsy (BP = Bell’s palsy and nipFP = non-idiopathic peripheral facial palsy) and healthy individuals (HI) showing a significant difference between the groups

Seventeen cytokines among patients with BP, nipFP, and HI were analyzed and compared. Regarding the levels of IL-1A, IL-5, IL-9, GM-CSF, CXCL10, Fract, MCP-1, IFN-y, IL-4, IL-17A, and TNF, no statistically significant difference between the three groups was found. However, significant differences were found for the cytokines G-CSF, CXCL13, TNFSF13, IL-8, IL-1ß and Granzyme: the levels of cytokines CXCL13, TNFSF13, and Granzyme were statistically significantly higher in patients with BP and nipFP compared to healthy individuals. The cytokine G-CSF was significantly higher in nipFP patients compared to HI and significantly higher if compared to BP patients. The level of cytokine IL-8 was significantly lower in both patient groups than in healthy individuals. The cytokine IL-1ß was significantly higher in nipFP patients compared to healthy individuals and patients with BP. The levels of the analyzed cytokines are shown in Table 2, and the significant differences are demonstrated in Fig. 2.

**Table 2:** Comparison of serum cytokines in facial palsy subtypes (BP and nipFP) and healthy individuals

Subtype of cytokine	Patients with BP Mean (SD) (ng/l)	Patients with nipFP Mean (SD) (ng/l)	Healthy individuals Mean (SD) (ng/l)	*p* value
*n*	40	7	10	
IL-1A	10 (11)	9.8 (9.6)	4.7 (4)	0.297
IL-5	2.1 (4.8)	1.1 (0.5)	1 (0.4)	0.642
IL-9	2.4 (2.1)	2.5 (1.9)	1.7 (0.5)	0.535
G-CSF	17.6 (17.3)	27.9 (9.6)	6.1 (4.5)	0.016
CXCL13	145 (70)	202 (120)	79 (22)	0.004
GM-CSF	1.53 (3.24)	1.98 (3.14)	0.84 (0.94)	0.716
TNFSF13	12626 (4580)	12317 (2354)	3449 (1031)	< 0.001
IL-8	13.7 (7.6)	17.3 (4.9)	431.5 (315.8)	< 0.001
IL-1ß	97.9 (98.7)	196.1 (187.9)	53.8 (38.3)	0.028
IP10 (CXCL10)	0.24 (0.6)	0 (0.01)	0.02 (0.05)	0.288
Fractalkine	32 (44.4)	41.9 (31.6)	9.3 (13)	0.249
MCP-1	109.4 (88.7)	160.4 (105.7)	165.4 (106.5)	0.149
IFN-y	1.32 (1.3)	1.53 (0.94)	0.82 (1.32)	0.454
IL-4	3.65 (6)	2.19 (2.13)	0.71 (0.44)	0.256
IL-17A	1.13 (1.29)	1.34 (0.78)	1.05 (0.54)	0.874
TNF	0.99 (1.31)	0.41 (0.55)	0.44 (0.28)	0.232
Granzyme	1.01 (0.68)	1.05 (0.49)	0.46 (0.15)	0.005

### Characteristics of cytokines in the CSF

**Fig. 3:**
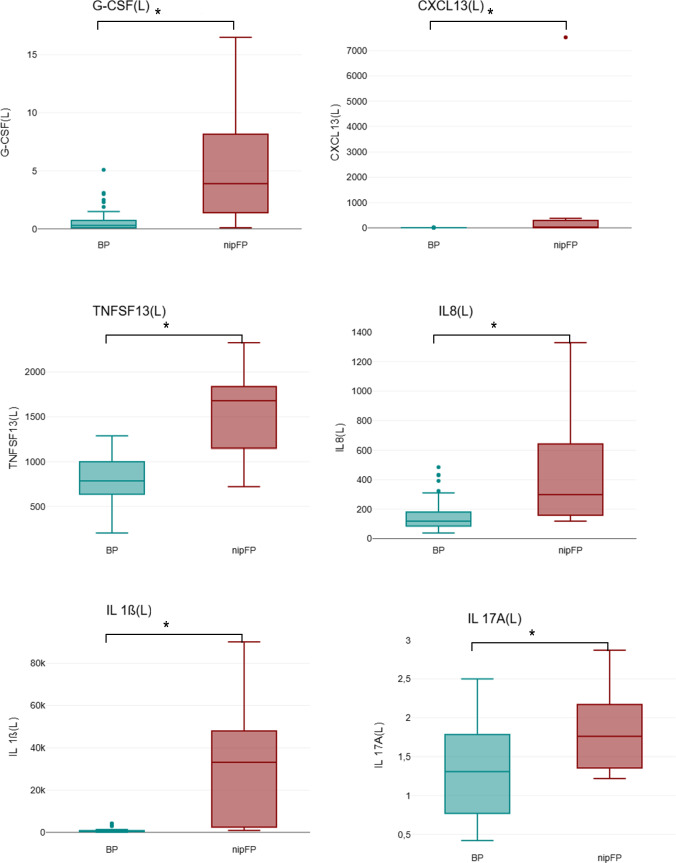
CSF (L) cytokine levels in patients with peripheral facial nerve palsy (BP = Bell’s palsy and nipFP = non-idiopathic peripheral facial palsy) showing a significant difference between the groups

The identical cytokines were also analyzed and compared in the cerebrospinal fluid of patients with BP and nipFP. Regarding the levels of IL-1A, IL-5, IL-9, GM-CSF, CXCL10, Fract, MCP-1, IFN-y, IL-4, TNF, and Granzyme, no statistically significant difference between the two groups was found. However, significant differences were found for the cytokines G-CSF, CXCL13, TNFSF13, IL-8, IL-1ß, and IL-17A; the levels of all these cytokines were statistically significantly higher in patients with nipFP compared to patients with BP. The levels of the analyzed cytokines of the CSF are shown in Table 3, and the significant differences are demonstrated in Fig. 3.

**Table 3:** Comparison of CSF cytokines in facial palsy subtypes (BP and nipFP)

Subtype of cytokine	Patients with BP Mean (SD) (ng/l)	Patients with nipFP Mean (SD) (ng/l)	*p* value
*n*	40	7	
IL-1A	1.4 (1.9)	6.1 (10)	0.27
IL-5	0.5 (0.26)	1.03 (0.83)	0.163
IL-9	2.3 (1.3)	2.8 (1.5)	0.30
G-CSF	0.75 (1.1)	5.66 (6.2)	0.007
CXCL13	6(2.5)	1326 (3040)	0.006
GM-CSF	0.23 (0.25)	0.35 (0.42)	0.294
TNFSF13	787 (263)	1529 (549)	0.011
IL-8	159 (114)	478 (437)	0.007
IL-1ß	835.6 (999.7)	32128.4 (33325.7)	0.048
IP10 (CXCL10)	0.13 (0.37)	0.26 (0.39)	0.42
Fractalkine	32.5 (25)	35.6 (18.7)	0.76
MCP-1	752.4 (645.2)	934 (695.9)	0.5
IFN-y	0.27 (0.32)	0.92 (1.04)	0.062
IL-4	0.23 (0.23)	0.21 (0.22)	0.847
IL-17A	1.33 (0.58)	1.84 (0.6)	0.037
TNF	0.23 (0.25)	0.35 (0.29)	0.276
Granzyme	1.1 (1.93)	19.3 (29.9)	0.159

### Correlation matrix of the cytokines

**Fig. 4:**
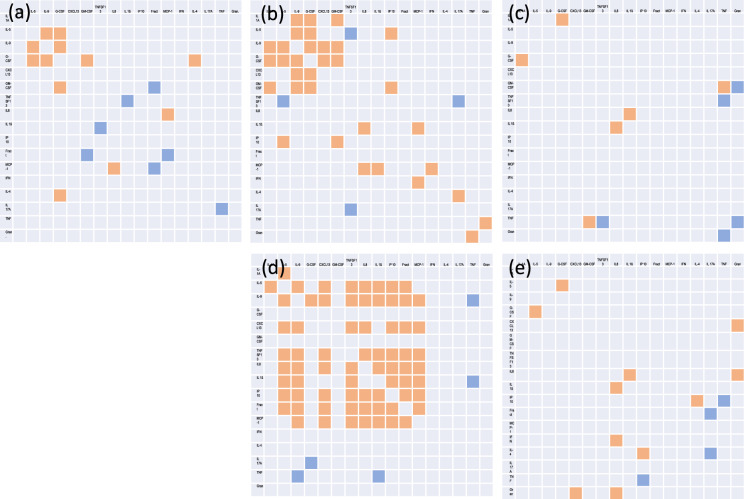
Heat map of the correlation matrix of the analyzed cytokines. **a** Serum, healthy individuals. **b** Serum patients with BP, **c** Serum patients with nipFP, **d** Cerebrospinal fluid patients with BP, **e** Cerebrospinal fluid patients with nipFP. (Orange fields indicate positive correlation, *p* < 0.05; blue fields indicate negative correlation, *p* < 0.05 (Spearman test)

The correlation matrix for the analyzed cytokines is presented in Fig. 4.

We found significant positive correlations much more frequently in patients with BP compared to healthy individuals or patients with nipFP in the serum and in the CSF. In the serum of patients with BP, the cytokines IL-1A, IL-9, GCSF, IL-5, CXCL 13 and GM-CSF formed a cluster. In the CSF of patients with BP, the cytokines IL-5, IL-8, Il-9, IL-1ß, TNFSF13, CXCL13, Fract, IP-10, and MCP-1 were cluster-like positively correlated.

### Diagnostic accuracy of serum cytokines

**Fig. 5:**
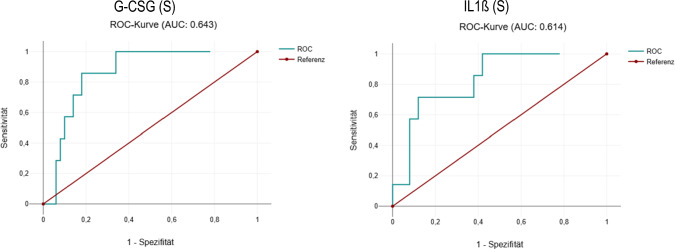
ROC curves of serum cytokine levels of G-CSF and IL-1ß differentiating patients with non-idiopathic peripheral facial palsy (nipFP) from Bell´s palsy (BP) showing a certain diagnostic accuracy

For differentiating nipFP from BP by serum cytokines alone, the AUC values of the serum cytokines G-CSF and IL1ß were 0.643 and 0.614, indicating a certain discrimination. All other serum cytokines showed no significant discrimination (Fig. 5).

### Correlation between severity of facial palsy and clinical outcome after 3 months and cytokines

The analysis of the association between the severity of facial palsy according to HB (grades II–III; grades IV–VI), serum, and CSF cytokines revealed an association between more severe facial palsy and higher levels of the CSF cytokine TNFSF13 (*p* = 0.02).

The analysis of the association between the clinical outcome after 3 months (improvement/no change) and serum and CSF cytokines revealed an association between more frequent unchanged facial palsy and higher levels of the CSF cytokine Fractalkine (*p* = 0.025).

### Correlation between CSF cell count and CSF cytokines

The analysis of the correlation between the CSF cell count and CSF cytokines in revealed a moderate positive correlation for CXCL13 (*r* = 0.37; *p* = 0.012), IL-8 (*r* = 0.33; *p* = 0.023), IL-1ß (*r* = 0.39; *p* = 0.006), IP-10 (*r* = 0.30; *p* = 0.042), IFN-a (*r* = 0.32; *p* = 0.042), and a strong correlation for granzyme (*r* = 0.57; *p* < 0.001).

## Discussion

Of the 17 cytokines tested, we found higher serum levels of G-CSF, CXCL13, TNFSF13, and Granzyme in patients with BP and nipFP, compared to healthy individuals. IL-1ß was higher in nipFP patients compared to BP patients and healthy individuals. Moreover, the level of cytokine G-CSF and IL-1ß was higher in nipFP patients than in BP patients. In simple terms, it could be said that these cytokines were elevated in patients with both BP and nipFP as an immunologic response. A further observation was that cytokines in patients with BP revealed a positive correlation with each other much more frequently. For differentiating nipFP from BP by serum cytokines, G-CSF and IL1ß indicated a slight discrimination. Patients with severe facial palsy (grades IV–VI according HB) revealed higher levels of the CSF cytokine TNFSF13, and clinical outcome after 3 months was less favorable at higher levels of the CSF cytokine Fractalkine. If the CSF cell count was elevated, higher levels of CXCL13, IL-8, IL-1ß, IP-10, IFNa, and granzyme in the CSF were found.

G-CSF is a proinflammatory cytokine that primarily supports the proliferation, differentiation, and survival of neutrophil granulocytes [15]. For example, elevated CSF G-CSF levels were found in neuromyelitis optica patients [16]. In our study, G-CSF was elevated in cerebrospinal fluid and serum and represented a certain discriminator. Higher levels distinguished partially patients with nipFP from patients with BP. CXCL13 is well known and elevated in neuroborreliosis and neurosyphilis [17, 18]. This cytokine is now used as a diagnostic tool for infections of the central nervous system and is also used to monitor therapy [19], but it is also increased in BP to a certain extent, as our study shows. Nevertheless, facial palsy, due to neuroborreliosis, may be a specific case with regard to the neuroinflammatory process, as in Lyme disease, even small amounts of bacterial agents, may lead to a complex orchestrated immune response [20]. TNFSF13 belongs to the TNF family and leads to the activation of B and T cells involved in inflammatory processes. It contributes to lymphocyte maturation and, in addition to physiological activities, can also develop pathological activities, such as tumor development or immunosuppression [21, 22]. Our findings of higher serum TNFSF 13 in both BP and nipFP patients and higher CSF TNFSF 13 in nipFP patients as well as the association of CSF TNFSF13 with more severe facial palsy could be indicative of an increased inflammatory activity mediated by B and T cells. Granzyme is known from rheumatology. It contributes to inflammation by degrading the extracellular matrix and promoting cytokine release [23]. Serum IL-1ß has recently be reported in the field of neurology to be associated with chronic cluster headache, and CSF IL-1ß was identified as an inflammatory biomarker for diagnosing bacterial meningitis [24, 25]. In our study, serum IL-1ß was graded elevated in both patient groups (higher in nipFP patients) and CSF IL-1ß was higher in nipFP patients. Furthermore, serum IL-1ß levels proved to be a discriminator, albeit a weak one, between BP and nipFP patients. IL-1ß is considered as a proinflammatory cytokine produced by monocytes, macrophages, and T cells. It induces the synthesis of acute phase proteins and increases the cytotoxicity of immune cells. IL-1ß also stimulates the production of IL-6 and influences IL-18 [7], neither of which were measured in our study. Hypothetically, IL-1ß could therefore be considered as a serum biomarker for distinguishing facial paralysis.

In contrast, the level of serum cytokine IL-8 was significantly lower in both patient groups than in healthy individuals. At a first glance, this finding is irritating. IL-8 is a pro-inflammatory cytokine that plays a role in several psycho-neuro-immunological processes and affects both neurological and psychiatric health as well as different various clinical conditions [26, 27]. It is produced by macrophages and stromal cells and develops antiviral, inflammatory, angio-genic, and pro-tumoral activities [7]. The main target of IL-8 action is the neutrophil granulocyte through the stimulation of their proliferation. High levels of IL-8 should have an unfavorable prognosis in both cancer and infectious diseases. However, in addition to IL-8 inducers, there are numerous IL-8 reducers such as other cytokines or even glucocorticoids [7]. Thus, the low serum Il-8 found can be influenced by the stage of immune response. Patients with facial palsy examined in this study are diagnosed in a very acute stage of immune response as facial palsy is easily and early visible and patients apparently come to clinic early. The test for IL-8 was therefore carried out at a very early stage although IL-8 is a sensitive marker of chronic inflammation. Another aspect could be that the low level found in our study indicates a favorable factor for healing facial palsy. The observed reduction in IL-8 may be a result of the increase of the abovementioned IL-8-reducing cytokines during the inflammatory process [26].

Fractalkine has chemo-attractive activity for monocytes, natural killer cells, and T cells and supports leukocyte adhesion. It can be induced by cytokines, such as TNF-α, IFN-γ, and IL-1ß, and it is involved in different diseases [28]. As it can be classified as a pro-inflammatory cytokine, it is conceivable that this cytokine in CSF is associated with delayed healing of facial nerve paralysis as found in our study. In a previous study, CSF fractalkine levels were significantly increased in patients with BP compared to controls and the fractalkine CSF/serum ratios tended to be increased. However, there was no evidence of significant intrathecal production of fractalkine [29].

The cytokine panels applied in our study were designed to interrogate innate and adaptive immune pathways. CXCL13 and TNFSF13 elevations point to B cell activation, while increased G-CSF and altered chemokines in nipFP indicate enhanced myeloid recruitment. These distinct profiles suggest a graded inflammatory spectrum between BP and nipFP, supporting hypotheses on infection-driven inflammation versus autoimmune neuritis [30, 31].

Only a few cytokine determinations in patients with BP are available to date. Yilmaz et al. (2002) detected elevated levels of IL-6, IL-8, and TNF-alpha in serum. As cytokines are not stored and performed in cells, these elevated cytokine levels are considered to represent a response to the underlying pathology in BP. However, whether the elevated cytokines also play a pathogenic role in BP is an open question [9]. In the study by Maxeiner et al., which included 10 patients with BP, higher levels of IL-8, TNF-alpha, slightly elevated IFN-y, IL-10 and IL-12 were found in the serum, and IL8 and IL-10 were measurable in the CSF indicating an immune response [10]. Experimentally, increased IL-2 and IL-4 were detected in HSV-1-induced facial nerve palsy as an indication of an immune response [32]. Elevated serum calprotectin levels also speak in favor of the inflammatory aspect of BP [33]. Analyses of the systemic immune-inflammation index also suggest a systemic inflammatory component [34]. Using the Mendelian randomization method, correlations between inflammatory proteins, metabolites, immune cells, and BP could be shown. The JAK/STAT signaling pathway, which is activated by cytokines, could play a special role and possibly be considered as a target for therapeutic intervention [35]. In the study by Masouris et al. (2023), BP patients with elevated CSF protein showed dysregulated proteins indicating an inflammatory response. In addition, the elevated cytokine levels found were similar to those in patients with facial palsy associated with the Varicella-zoster virus. The authors discuss an upregulated inflammatory pathway for patients with BP and elevated protein in the cerebrospinal fluid [11].

The great difficulty in evaluating these studies is the marked heterogeneity of the investigations. However, all these studies conclude that inflammatory and immunological processes are recognizable by a certain cytokine profile. In this sense, our study contributes to the detection of certain cytokines in patients with BP, as the increase in cytokines G-CSF, CXCL13, TNFSF13, Granzyme and IL-1ß, as well as the decrease in cytokine IL-8, which has not yet been reported.

Can our study contribute to the etio-pathogenesis of BP? Our cytokine findings do not conclusively prove either HSV reactivation or a purely cell-mediated autoimmune mononeuritis, but they provide mechanistic hints consistent with a mixed pathogenic spectrum. The elevation of CXCL13 and TNFSF13 suggests B cell recruitment and survival, processes commonly observed in CNS infections and B cell–driven neuro-inflammation. Higher G-CSF levels and broader CSF cytokine elevations in pathogen-associated facial palsy point to stronger innate and myeloid activation typical of infection-related inflammation. In contrast, the increase in Granzyme A indicates cytotoxic effector engagement, a mechanism that can occur in both antiviral responses and autoimmune cytotoxicity, and therefore is not uniquely discriminative. Taken together, these results favor an overlapping model in which infection-triggered and immune-mediated mechanisms coexist: non-idiopathic facial palsy shows a stronger myeloid/CSF inflammatory signature, whereas idiopathic Bell’s palsy demonstrates coordinated lymphoid and effector signals. This interpretation supports the concept that BP represents a neuro-immunological continuum rather than a strict dichotomy between viral and autoimmune pathogenesis.

Several limitations of our study must be acknowledged. First, the number of patients with nipFP is relatively small, and the differences must therefore be carefully evaluated. Second, the age of the healthy control subjects is younger than that of the patients, so a potential age bias cannot be exactly ruled out. Third, of the numerous cytokines known to date [7], only 17 have been analyzed. No statements can therefore be made about the remaining cytokines. However, a selection of cytokines is unavoidable due to financial resources, chemical determination, and measurement methods. Fourth, no CSF analysis was available for healthy individuals, meaning that a comparison of CSF cytokines between patients with idiopathic facial nerve palsy and healthy individuals could not be carried out. And fifth, we did not perform systematic virologic PCR or detailed cellular immunophenotyping, which limits definitive attribution of cytokine signatures to viral reactivation versus autoimmune mechanisms.

In conclusion, compared to healthy individuals, our pilot study revealed an altered cytokine profile in patients with BP that resembles patients with nipFP. In CSF, a subset of cytokines was identified in patients with BP, but higher levels were found in patients with nipFP, suggesting a graduated inflammatory process.

## Data Availability

The full dataset and statistical analysis will be available upon reasonable request.

## Abbreviations

IL-1A: Interleukin 1 alpha
IL-5: Interleukin 5
IL-9: Interleukin 9
G-CSF: granulocyte colony-stimulating factor
CXCL13: C-X-C motif chemokine ligand 13
GM-CSF: Granulocyte–macrophage colony-stimulating factor
TNFSF13: Tumor necrosis factor ligand superfamily member 13 (APRIL)
IL-8: Interleukin 8
IL-1β: Interleukin 1 beta
IP-10: Interferon gamma-inducible protein 10
Fractalkine: Chemokine (C-X-3-C motif) ligand 1
MCP-1: Monocyte chemoattractant protein 1
IFN-γ: Interferon gamma
IL-4: Interleukin 4
IL-17A: Interleukin 17A
TNF: Tumor necrosis factor alpha
Granzyme A: Granzyme A
